# Increased Nuchal Translucency in Fetuses With a Crown-Rump Length of < 45 mm: Clinical Outcomes and Diagnostic Implications

**DOI:** 10.7759/cureus.103386

**Published:** 2026-02-10

**Authors:** Philipp Kreiselmaier, Makis Papadopolous, Jochen Ritgen, Jan Degenhardt

**Affiliations:** 1 Centre of Prenatal Medicine, Amedes Experts, Hamburg, DEU; 2 Centre of Prenatal Medicine and Genetics, Praenatal Plus, Cologne, DEU; 3 Centre of Prenatal Medicine, Klinikum Dortmund, Hospital of Witten/Herdecke University, Dortmund, DEU

**Keywords:** first-trimester pregnancy, miscarriage, nuchal translucency, pregnancy outcome, risk assessment

## Abstract

Background/Objectives: Nuchal translucency (NT) measurement is an established first-trimester screening tool for chromosomal abnormalities. However, standardized risk assessment models are only validated for fetuses with a crown-rump length (CRL) between 45 and 84 mm. This study investigates the clinical significance of increased NT in fetuses with a CRL between 35 and 45 mm.

Methods: This retrospective study analyzed 208 singleton pregnancies with a CRL < 45 mm and an NT ≥ 2.5 mm, examined at two tertiary prenatal centers between 2008 and 2024. Data on chromosomal findings, structural anomalies, and pregnancy outcomes were collected through follow-up. Statistical analyses included univariate, bivariate, and receiver operating characteristic curve analysis to determine optimal NT cut-off values for predicting adverse outcomes.

Results: Chromosomal abnormalities were present in 67/208 cases (32.2%), with trisomy 18 being the most frequent. Malformations were detected in 46.7% of cases with available follow-up. The optimal NT cut-off for predicting an abnormal karyotype was 3.89 mm (sensitivity 79.1%, specificity 55.3%). Among fetuses with NT ≥ 5.5 mm, the rate of adverse outcomes reached 83.7%. Notably, 29.9% of chromosomal abnormalities would not have been detected by cell-free DNA screening for aneuploidy alone. Overall, the presence of increased NT, even below the standard CRL threshold, was associated with a significantly elevated risk of genetic and structural abnormalities as well as adverse pregnancy outcomes.

Conclusions: NT measurement in fetuses with CRL < 45 mm provides clinically relevant prognostic information. Elevated NT in this early gestational window is significantly associated with chromosomal anomalies, structural defects, and poor pregnancy outcomes. Reliance on cell-free DNA screening for aneuploidy alone in this cohort may miss a substantial proportion of abnormal cases. Early NT assessment should prompt comprehensive diagnostic evaluation and genetic counseling, even when the CRL is below 45 mm.

## Introduction

Nuchal translucency (NT) measurement for risk calculation of chromosomal abnormalities is an established procedure. However, risk calculation is only possible with a fetal crown-rump length (CRL) of 45mm to 84mm. Due to the German maternity guidelines, the first ultrasound screening takes place between the 9th and 11th weeks of pregnancy. Therefore, there are repeatedly pregnant women whose fetuses show increased NT and for whom no risk calculation can be performed due to a CRL < 45mm. In this study, we have evaluated the outcome in cases of increased NT and a CRL between 35mm and 45mm. NT is defined as the maximum thickness of the subcutaneous translucent space between the skin and soft tissue overlying the cervical spine at the back of the fetal neck, observed during an ultrasound examination in the first trimester. Normal NT values lie below the 95th percentile for a given CRL, corresponding to approximately ≤2.5-3.0 mm in early first-trimester fetuses. Elevated NT measurements have been correlated with chromosomal and congenital abnormalities [1,2]. Vigilant assessment is critical for fetuses exhibiting increased NT, especially when coupled with other abnormalities [3].

The observation of increased NT in first-trimester fetuses, particularly those with a CRL of 45 mm or less, presents a diagnostic and management challenge in prenatal care, necessitating a comprehensive evaluation to ascertain potential underlying etiologies and inform subsequent clinical decisions [4]. Increased NT, identified through ultrasound, has been associated with a spectrum of fetal aneuploidies, structural anomalies, and genetic syndromes [5]. While NT measurement is a routine component of first-trimester screening for chromosomal abnormalities, its interpretation in fetuses with a CRL less than 45 mm requires careful consideration due to the limitations of established risk calculation models. NT screening is used in the first trimester to identify pregnancies at high risk for chromosomal abnormalities such as Down syndrome [6]. Early identification of increased NT allows for timely parental counseling, comprehensive diagnostic evaluation, and informed decision-making regarding pregnancy management.

The primary objective of this study was to evaluate the prognostic value of increased NT in fetuses with a CRL <45 mm. Secondary objectives included the assessment of associated chromosomal abnormalities, structural malformations, adverse pregnancy outcomes, and the modeled limitations of cfDNA-based screening in this early gestational window.

A previous version of the article was posted as a preprint in August 2025 [7].

## Materials and methods

Study design and patient selection, data collection, and analysis

The present work is a retrospective multi-center cohort study. Data were collected from two tertiary referral centers. The patients presented themselves at their own request for first-trimester screening or were referred by their gynecologist. The data refer to cases that were examined in the period from February 2008 to February 2024. The datasets were created using the program VIEWPOINT (GE Healthcare, Solingen, Germany) or ASTRAIA (Nexus/Astraia, Ismaning, Germany). The indications for the examination were the exclusion of conspicuous NT or fetal pathology, advanced maternal age, maternal anxiety, family history, and pregnancies with genetic or structural abnormalities. Inclusion criteria for data collection were singleton pregnancy, CRL < 45mm and NT ≥ 2.5mm. The study population consists of 208 cases that met these inclusion criteria. All data were stored in two computer-aided databases, Astraia and Viewpoint. Information about the outcome of the pregnancy was provided by the gynecologists, the maternity clinics, or the patients. NT was measured according to the guidelines of the Fetal Medicine Foundation (FMF), based on the gestational week and CRL [8]. A detailed ultrasound examination was performed on all fetuses to identify other structural anomalies. All ultrasound examinations were performed using state-of-the-art high-end ultrasound systems available at the time of the examination (GE Voluson 730 Expert, GE Voluson E8, GE Voluson E10, GE Voluson Expert 22). All ultrasound examinations were performed by qualified DEGUM II or DEGUM III certified examiners (Deutsche Gesellschaft für Ultraschall in der Medizin, DEGUM). All patients underwent genetic counseling with the option for invasive diagnostic testing (chorionic villus sampling or amniocentesis). Karyotype was determined through invasive diagnostic testing, NIPT (Non-invasive Prenatal Test, NIPT) testing, a combination of the two, or genetic examination of the aborted material.

The primary objective of the study was to evaluate the clinical significance of increased NT in fetuses with a CRL <45 mm. As a secondary objective, we assessed the proportion of chromosomal abnormalities that would not have been detected by standard NIPT, based on the spectrum of abnormalities identified in our cohort.

Statistical analysis 

The collected data were initially documented in a Microsoft Excel. After collection, it was anonymized and encrypted for storage. The Excel spreadsheet listed the maternal age, CRL, NT, the name of the examiner, the gestational age, the karyotyping or results of the fetal genetic examination, the outcome, the presence of malformations, the induced abortion, and the occurrence of a miscarriage or intra-uterine fetal demise (IUFD). A composite endpoint combining abnormal karyotype, structural malformations, and adverse pregnancy outcome was defined to capture overall clinically relevant adverse outcomes associated with increased NT. In addition, each component of the composite endpoint was analyzed separately to assess differential associations.

Data were analyzed using IBM SPSS Statistics for Windows, Version 27 (Released 2020; IBM Corp., Armonk, New York, United States). Descriptive statistics were used to summarize maternal and fetal parameters (univariate analysis). Bivariate analyses were performed to assess associations between NT measurements and different outcomes, including chromosomal abnormalities, structural malformations, and adverse pregnancy outcomes. For each outcome, only cases with available data were included in the respective analyses. Categorical variables were compared using Pearson’s chi-squared test or Fisher’s exact test, as appropriate. Receiver operating characteristic (ROC) analysis was performed to evaluate the performance of NT (mm) as a continuous predictor of adverse outcomes and to determine optimal cut-off values. 

All p-values are results of two-sided tests. A p-value below the significance level of p<0.05 was considered significant. 

## Results

Age distribution 

The cohort displayed a wide range of ages. The youngest patient was 19 years old, and the oldest was 49 years old. The mean age of the patient population was 32 years. The majority of cases, at 29.3%, were in the age group between 31 and 35 years. The age groups under 20 years and over 45 years each represented a small percentage of 2.4% and 0.5%, respectively.

Crown-rump-length

At the time of the examination, the CRL ranged from 23.2 to 44.9 mm, with a mean of 40.0 mm. The majority of fetuses had a CRL between 40.0 and 44.9 mm.

Nuchal translucency

The average NT thickness was 4.4 mm. The range was between 2.5 and 9.1 mm. The NT was between 2.5 and 3.5 mm with 30.3%, between 3.5 and 4.5 mm with 30.3%, between 4.5 and 5.5 mm with 17.3%, and greater than or equal to 5.5 mm with 22.1%.

Karyotype

**Figure 1:**
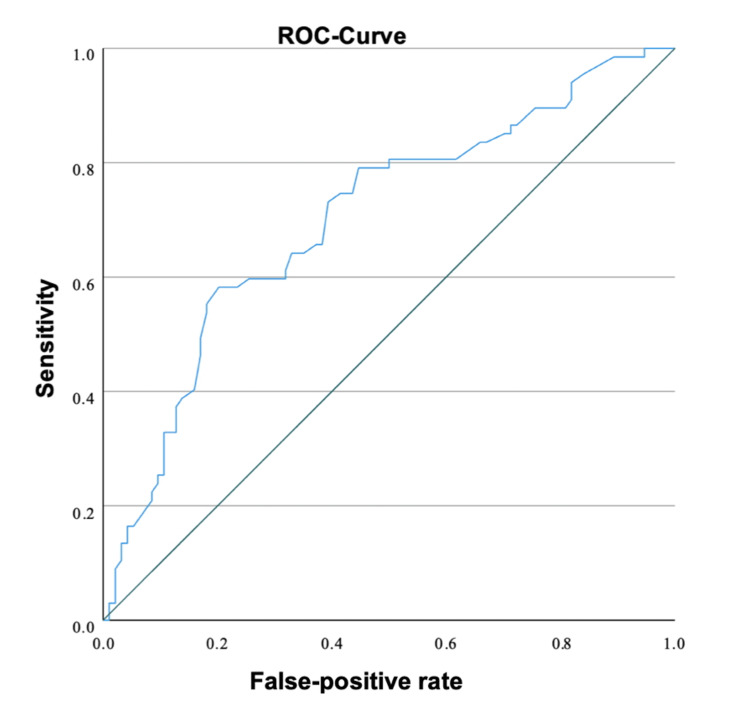
Receiver operating characteristic (ROC) curve of nuchal translucency for the prediction of chromosomal abnormalities ROC analysis demonstrated a moderate discriminative ability of NT for chromosomal abnormalities (AUC 0.69, 95% CI 0.61–0.77). NT: Nuchal translucency

Of the 208 cases examined, 67 had chromosomal abnormalities. Ninety-four fetuses showed no abnormalities. For the remaining 47, the karyotype was not determined, or it is unknown whether a genetic examination was performed. Among the chromosomally abnormal fetuses, 32 had trisomy 18, 13 had monosomy X, 11 had trisomy 21, four had trisomy 13, and seven had other chromosomal anomalies (Table 1, Table 2). An increased NT was strongly associated with abnormal karyotypes. Dichotomizing NT values yielded odds ratios (ORs) of 2.62 (1.25-5.49) for NT ≥3.5 mm, 5.28 (2.65-10.52) for NT ≥4.5 mm, and 5.72 (2.74-11.94) for NT ≥5.5 mm (all p < 0.001). The best cut-off value is 3.9 mm. At this cut-off value, the sensitivity is 79.1% and the specificity is 55.3% (Figure 1).

**Table 1:** Study characteristics SD: Standard deviation; CRL: Crown-rump-length; NT: Nuchal translucency; NIPT: Non-invasive prenatal test

Parameter		Mean ± SD / %
CRL		39.95 (3.47)
NT		4.40 (1.4)
Gestational Age (days)		76.45(4.65)
NT	2.5-3.5mm	63 (30.3%)
	3.5-4.5mm	63 (30.3%)
	4.50-5.5mm	36 (17.3%)
	≥ 5.5mm	46 (22.1%)
Karyotype	Missing	47 (22.6%)
	Normal	94 (45.2%)
	Trisomy 18	32 (15.4%)
	Trisomy 13	4 (1.9%)
	Trisomy 21	11 (5.3%)
	Monosomy X	13 (6.3%)
	Structural chromosomal abnormality	3 (1.4%)
	Triploidy	3 (1.4%)
	Trisomy 9	1 (0.5%)
Invasive Testing and/or NIPT	No	45 (21.6%)
	CVS	150 (72.1%)
	Amniocentesis	2 (1.0%)
	NIPT	4 (1.9%)
	Unknown	6 (2.9%)
	CVS and Amniocentesis	1 (0.5%)
Aneuploidy	No	94 (45.2%)
	Yes	64 (30.8%)
	Unknown/Missing	50 (24.0%)
Termination of Pregnancy	No	106 (51.0%)
	Yes	56 (26.9%)
	Unknown/Missing	46 (22.1%)
Malformation	No	65 (31.3%)
	Yes	57 (27.4%)
	Unknown/Missing	86 (41.3%)
Cardiac Anomalies	No	68 (32.7%)
	Yes	36 (17.3%)
	Unknown/Missing	104 (50%)
Abnormal soft markers beyond other than NT	No	96 (46.2%)
	Yes	74 (35.6%)
	Unknown/Missing	38 (18.3%)

**Table 2:** NT and chromosomal aberrations Data are shown as n (%) NT: Nuchal translucency

NT	Normal Karyotype	Trisomy 18	Trisomy 13	Trisomy 21	Monosomy X	Structural Chromosomal Anomalies	Triploidy	Trisomy 9
2.5-3.5mm	32 (74.4%)	4 (9.3%)	1 (2.3%)	3 (6.9%)	2 (4.7%)	0 (0.0%)	0 (0.0%)	1 (2.3%)
3.5-4.5mm	35 (68.6%)	5 (9.8%)	1 (2.0%)	6 (11.8%)	0 (0.0%)	2 (3.9%)	2 (3.9%)	0 (0.0%)
4.5-5.5mm	15 (48.4%)	12 (38.7%)	0 (0.0%)	0 (0.0%)	3 (9.7%)	1 (3.2%)	0 (0.0%)	0 (0.0%)
≥5.5mm	12 (33.3%)	11 (30.5%)	2 (5.6%)	2 (5.6%)	8 (22.2%)	0 (0.0%)	1 (2.8%)	0 (0.0%)
Total	94 (58.4%)	32 (19.9%)	4 (2.5%)	11 (6.8%)	13 (8.1%)	3 (1.9%)	3 (1.9%)	1 (0.6%)

Malformations

In 122 cases, complete information on a malformation was available. In 57 cases, a malformation was present, and in 65 cases, it was not. The risk of structural malformations increased significantly with higher NT. Compared to NT 2.50-3.49 mm, the odds were 1.30 (95 % confidence interval (CI) 0.49-3.45) for NT 3.50-4.49 mm, 5.83 (1.84-18.47) for NT 4.50-5.49 mm, and 7.92 (2.55-24.60) for NT ≥5.5 mm. Dichotomizing NT showed ORs of 2.74 (1.17-6.43) for NT ≥3.5 mm, 6.48 (2.80-15.02) for NT ≥4.5 mm, and 7.38(3.03-17.96) for NT ≥5.5 mm (all p < 0.001). The cut-off value of the NT with the highest specificity and sensitivity is 3.9 mm. At this cut-off value, the sensitivity is 68.4% and the specificity is 69.2%.

Malformations and/or karyotype

Complete information on malformations and/or karyotype was available for 150 cases. In 100 cases, one or both parameters were abnormal, and in 50 cases, both were known to be normal. In 58 cases, both parameters were missing or one parameter was normal and the other was missing. In 100 cases, a malformation and/or an abnormal karyotype was present, and in 50 cases, it was not. The combined risk of structural malformations and/or abnormal karyotype increased significantly with higher NT. Compared to NT 2.50-3.49 mm, the odds were 1.64 (95 % CI 0.52-5.13) for NT 3.50-4.49 mm, 3.14 (0.92-10.66) for NT 4.50-5.49 mm, and 10.18 (2.98-34.73) for NT ≥5.5 mm. Dichotomizing NT showed ORs of 3.44 (1.36-8.69) for NT ≥3.5 mm, 6.71 (2.73-16.49) for NT ≥4.5 mm, and 12.08 (4.57-31.90) for NT ≥5.5 mm (all p < 0.001). The cut-off value of the NT with the highest specificity and sensitivity is 3.6 mm. At this cut-off value, the sensitivity is 78.0% and the specificity is 52.0%. Tables 3-5 summarize absolute numbers, percentages, and ORs with 95% CIs for abnormal karyotype, structural malformations, and the combined endpoint (malformation and/or abnormal karyotype) across different NT categories. 

**Table 3:** Abnormal karyotype according to NT categories Outcomes across four NT subgroups. Odds ratios (ORs) with 95% confidence intervals (CIs) are provided relative to the reference category (2.50–3.49 mm). NT: Nuchal translucency

NT category	Abnormal karyotype, n (%)	OR vs Ref (95% CI)	p-value
2.50–3.49 mm	11/43 (25.6%)	1.00 (Ref)	<0.001
3.50–4.49 mm	16/51 (31.4%)	1.33 (0.55–3.20)	
4.50–5.49 mm	16/31 (51.6%)	3.11 (1.22–7.91)	
≥ 5.50 mm	24/36 (66.7%)	5.82 (2.29–14.81)	

**Table 4:** Fetal malformations according to NT categories Outcomes across four NT subgroups. Odds ratios (OR) with 95% confidence intervals (CIs) are provided relative to the reference category (2.50–3.49 mm). NT: Nuchal translucency

NT category	Malformation, n (%)	OR vs Ref (95% CI)	p-value
2.50–3.49 mm	12/42 (28.6%)	1.00 (Ref)	<0.001
3.50–4.49 mm	12/35 (34.3%)	1.30 (0.49–3.45)	
4.50–5.49 mm	14/20 (70.0%)	5.83 (1.84–18.47)	
≥ 5.50 mm	19/25 (76.0%)	7.92 (2.55–24.60)	

**Table 5:** Abnormal karyotype and/or fetal malformation according to NT categories Outcomes across four NT subgroups. Odds ratios (ORs) with 95% confidence intervals (CIs) are provided relative to the reference category (2.50–3.49 mm). NT: Nuchal translucency

NT category	Abnormal karyotype and/or malformation, n (%)	OR vs Ref (95% CI)	p-value
2.50–3.49 mm	7/18 (38.9%)	1.00 (Ref)	<0.001
3.50–4.49 mm	23/45 (51.1%)	1.64 (0.52–5.13)	
4.50–5.49 mm	18/27 (66.7%)	3.14 (0.92–10.66)	
≥ 5.50 mm	52/60 (86.7%)	10.18 (2.98–34.73)	

Pregnancy outcome

The outcome is known for 164 cases. The term "negative outcome" refers to cases with a miscarriage, a termination of pregnancy, an IUFD, or postnatal death within the first three months after birth. In 74 cases, there was a positive outcome, and in 90 cases, there was not. The rate of adverse outcomes was significantly higher in fetuses with NT ≥ 3.9 mm (74.4%) compared to those with NT < 3.9 mm (25.6%, χ² test, p < 0.001).

**Figure 2:**
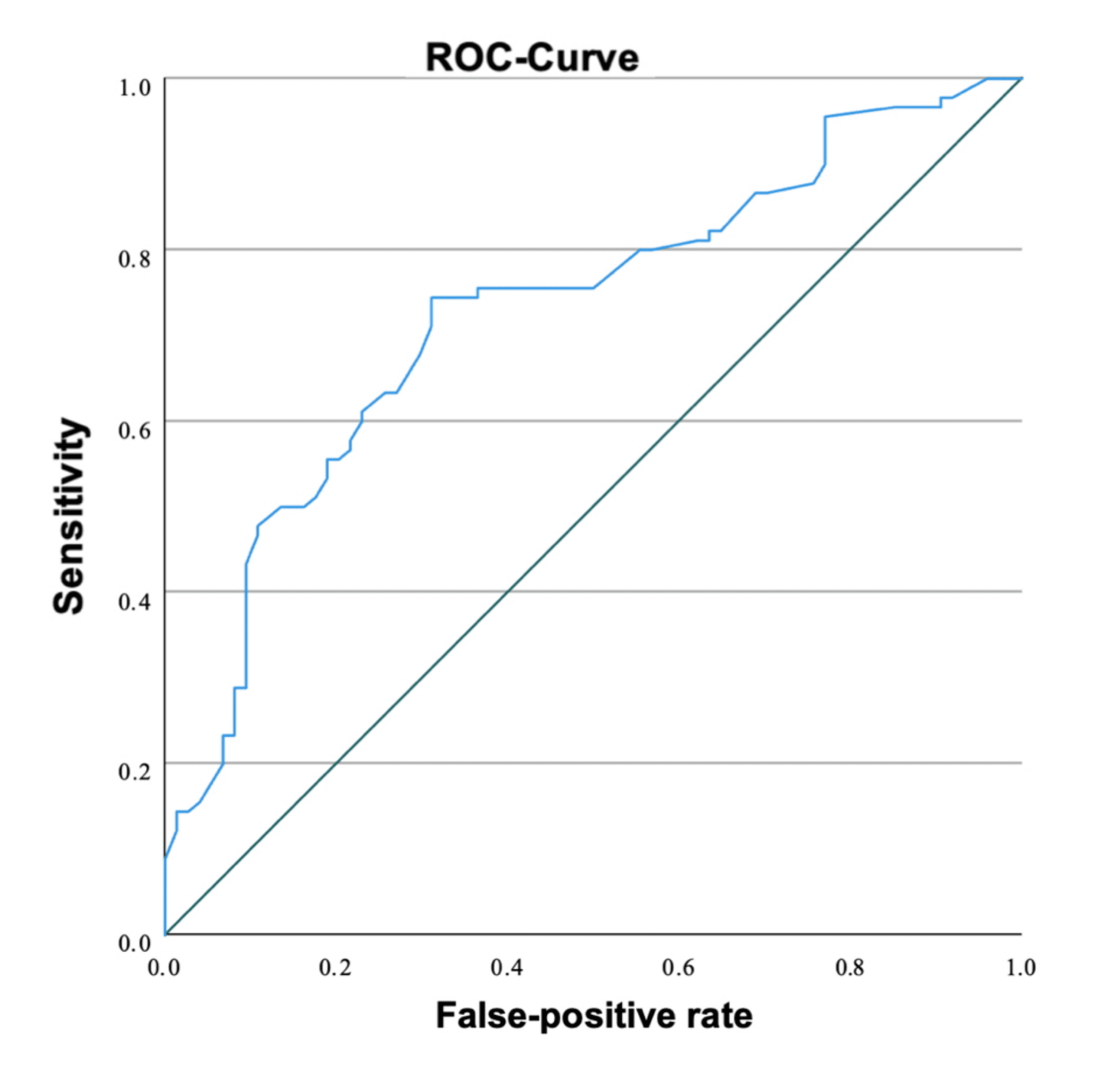
Receiver operating characteristic (ROC) curve of nuchal translucency for the prediction of adverse pregnancy outcome ROC analysis demonstrated a moderate discriminative ability of nuchal translucency NT for adverse pregnancy outcome, with an AUC of 0.74 (95% CI 0.65–0.84). NT: Nuchal translucency

**Figure 3:**
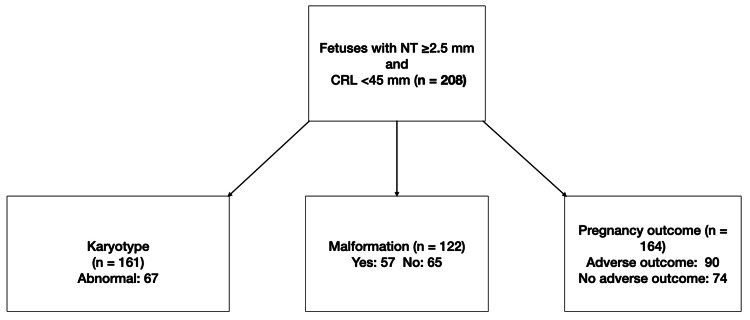
Study flow diagram. Overview of case inclusion and outcome data Due to the retrospective study design, availability of karyotype, malformation, and pregnancy outcome data differed across cases. NT: Nuchal translucency; CRL: Crown-rump length

ROC analysis (Figure 2) confirmed that an NT cut-off of 3.9 mm provided the best discrimination between positive and negative outcomes, yielding a sensitivity of 74.4% and a specificity of 68.9%. The study flow diagram is shown in Figure 3.

Negative outcomes and/or abnormal karyotype and/or malformations

For 178 cases, the information on a malformation and/or abnormal karyotype and/or negative outcome was complete. In 104 cases, at least one of the above-mentioned abnormalities was present, and in 74 cases, it was not. The best cut-off value is 3.89. At this cut-off value, the sensitivity is 76.0% and the specificity is 68.9%. Among the 46 fetuses with NT ≥ 5.5 mm, 39 cases (83.7%) were associated with at least one abnormal finding, including chromosomal abnormalities, structural malformations, or adverse pregnancy outcomes. 

NIPT 

NIPT, which has been a statutory health insurance benefit in Germany since 2022, is used to identify trisomies 13, 18, and 21. In our cohort, NIPT was performed in only four cases. To evaluate its potential diagnostic performance, we compared the spectrum of chromosomal abnormalities identified in our study (n = 67) with the conditions routinely covered by standard NIPT (trisomies 13, 18, and 21).

Among the 67 fetuses with chromosomal abnormalities, 29.9% (n = 20) had aberrations that would not have been detected by currently available standard NIPT, including monosomy X (n = 13), structural chromosomal defects (n = 3), triploidy (n = 3), and trisomy 9 (n = 1) (Table 4). Among the 104 cases with an adverse outcome, malformation, and/or abnormal karyotype, only 56 cases (54%) would have been identified by standard NIPT, which targets trisomies 13, 18, and 21. The remaining 48 cases (46%) involved chromosomal or structural abnormalities not detectable by standard NIPT. These results therefore refer to the entire subgroup of fetuses with abnormal karyotypes, not just those who underwent NIPT.

## Discussion

Our data show that an increased NT is also associated with an elevated probability of an adverse outcome in fetuses with a CRL < 45 mm. The likelihood of a malformation and/or an abnormal karyotype increases from 24.0% for NT between 2.5 mm and 3.9 mm to 76.0% for NT equal to or greater than 3.9 mm. Furthermore, we were able to demonstrate that if only NIPT were performed, only 54% of cases with an adverse outcome and/or malformation and/or abnormal karyotype would have been diagnosed. The guidelines state that NT measurement should be performed as part of first-trimester screening, with a CRL between 45 mm and 84 mm. These thresholds have been modified several times by the Nicolaides group until a specific time interval was finally defined in 2012. The frequency of chromosomal anomalies was mainly investigated for CRL values between 45 mm and 84 mm [9-11]. The question arose whether assessment in earlier weeks of pregnancy and shorter CRL can have equivalent diagnostic accuracy. Our data show that the majority of pregnancies with an increased NT > 2.5 mm have a poor prognosis. Of 178 cases where the information on a malformation and/or abnormal karyotype and/or negative outcome was complete, it was found that in 104 cases at least one of the abnormalities was present. In cases with an NT > 5.5 mm, the probability of a poor prognosis is 83.7%. 

Pandya et al. showed that the more widespread the NT, the higher the risk of chromosomal abnormalities. In this study, where NT was measured in fetuses between 10 and 14 weeks of gestation, the observed number of trisomies 13, 18, and 21 in fetuses with a NT of 3 mm, 4 mm, 5 mm, and > 6 mm was approximately 3 times, 18 times, 28 times, and 36 times higher than the corresponding number expected based on maternal age [12].

Given that a significantly increased NT is a risk factor for the presence of malformations or chromosomal anomalies, it was to be expected that this would be similar in our study. While in the groups with NT 2.5-3.5 mm and 3.5-4.5 mm, the karyotype was abnormal in 25.6% and 31.4%, respectively, in the groups with NT 4.5-5.5 mm and > 5.5 mm, it was 51.6% and 66.7%, respectively.

In our study, the karyotype could be determined in 161 cases. Ninety-four cases showed a normal karyotype, while 67 cases had an abnormal karyotype (41.6%). These values are similar to the data from Lugthart et al., where the proportion of fetuses with a normal karyotype was 54.2% [13]. In the study by Grande et al., the proportion of fetuses with an abnormal karyotype was only 7.2% [14]. This is explained by stricter inclusion criteria in both our study model and the Lugthart study model, which, in addition to a CRL < 45 mm, also included NT as an inclusion criterion.

Our findings align with literature regarding the prevalence of certain chromosomal anomalies, trisomies 13, 18, and 21, with trisomy 18 being the most common. The most frequently detected trisomy in pregnancy is trisomy 21, while in our study, as well as in the studies by Lugthart et al. and Grande et al., trisomy 18 was most common [13,14]. One possible explanation could be that some fetuses with trisomy 18 had already miscarried by the time of the actual first-trimester screening later in pregnancy, thereby lowering the observed prevalence.

In our study, the most significant cut-off value for detecting the abnormal karyotype was 3.9 mm. In fact, 20.9% of cases with an abnormal karyotype had an NT of less than 3.9 mm. This is a high percentage, but the Brandi study, with a cut-off of 3.5 mm, the most common cut-off for further genetic tests, showed that 23.4% of embryos with anomalies had an NT of less than 3.5 mm. The established NT thresholds for CRL measurements above 45 mm do not accurately predict risks in pregnancies at early gestational stages. The NT measurement of 3.9 mm in our study population demonstrates equivalent predictive value to the established thresholds used during later gestational periods, which indicates that first-trimester screening can deliver useful clinical information. The results confirm that healthcare providers should refer patients with increased NT measurements to specialized fetal medicine units and perform complete anatomical ultrasounds and genetic counseling, even when CRL measurements fall below the typical range.

Our study demonstrated that 29.9% of fetuses had chromosomal abnormalities that would not have been detected by NIPT. This deserves particular emphasis, as the increasing adoption of NIPT as a first-line screening tool must not overshadow the importance of the early fetal anomaly scan in combination with expert genetic counseling and diagnostic evaluation in cases of abnormal findings.

Numerous studies have shown that an increased NT is associated not only with an increased risk of chromosomal anomalies, but also with an increased risk of malformations, even when the karyotype is normal [15-18]. In our study, complete information on a malformation was available in 122 cases. In 57 cases, a malformation was diagnosed. In 33 cases with malformations, the NT was > 4.5 mm. This finding supports previous reports demonstrating that the risk of congenital malformations rises disproportionately with increasing NT thickness, irrespective of chromosomal status.

Notably, the risk of an adverse outcome was significantly higher when NT exceeded 4.5 mm, with 55.6% of cases with an adverse outcome in this group. Spataro et al. found that in fetuses with an NT of ≥ 3.5 mm and a normal karyotype, the rate of adverse outcome was directly correlated with the NT width [19]. In summary, the data indicate a direct association between the NT and pregnancy outcome, irrespective of the presence of genetic anomalies. Our study focused on the detection of chromosomal abnormalities using karyotyping, NIPT, or genetic testing of products of conception. We did not systematically investigate other types of genetic disorders, such as single-gene mutations or copy number variations, since these analyses were not routinely available during a large part of the rather long study period. It should be noted that comprehensive molecular genetic testing, including exome sequencing or targeted gene panels, has only become widely accessible in Germany in recent years, particularly since these techniques have been covered by statutory health insurance. Therefore, our conclusions regarding ‘genetic anomalies’ are limited to chromosomal findings.

Bergsch et al. observed that the 'Radiant' image enhancement mode in modern ultrasound systems significantly increases measured NT values. These recent advancements in ultrasound imaging highlight the need to reevaluate existing NT reference charts. As the debate continues over appropriate NT cutoff values, whether 3.0 mm, 3.5 mm, or the 99th percentile, such technological factors must be considered, particularly since these thresholds closely correspond to those used in current first-trimester screening protocols [20]. These findings underline the importance of considering technological factors when establishing NT reference charts and determining appropriate cut-off thresholds, particularly since current first-trimester screening protocols still rely heavily on fixed NT cut-offs (e.g., 3.0 mm, 3.5 mm, or the 99th percentile). Future studies should aim to stratify NT values according to the type and generation of ultrasound equipment to better understand the impact of image enhancement technologies on NT measurement.

The results of this study demonstrate the clinical utility of NT measurement even when the CRL is less than 45 mm, suggesting that elevated NT measurements at early gestational stages are indicative of potential adverse outcomes. The management of patients presenting with an elevated NT at an early gestational age, specifically prior to 11 weeks, can be difficult, as studies have proven that an initial increase in NT followed by normalization is a common finding [21,22]. In counseling expectant parents, it is essential to emphasize that, despite the potential for structural or genetic abnormalities that may be diagnosed at a later stage, the likelihood of delivering a healthy child remains a realistic possibility. Performing NIPT in this particular patient group does not provide additional benefit, as the detectable spectrum of conditions remains too limited, at least with currently available regulations. These estimates should be interpreted with caution, as they are derived from a modeled comparison between observed karyotypes and the theoretical detection range of cfDNA-based screening, rather than from direct test performance within this cohort.

Although this study investigates fetuses in a very early gestational window, prior to routine first-trimester screening, the cohort represents a selected referral population. Therefore, the prevalence of abnormalities and the predictive performance of NT may not be directly generalizable to unselected routine screening populations. In addition, this study has further limitations that should be considered when interpreting the findings. First, its retrospective design may have introduced selection bias, as cases were recruited from two tertiary referral centers. Second, the study period spans 2008 to 2024, during which diagnostic technologies evolved considerably. Advanced image optimization modes, such as “Radiant” enhancement, became available only in the later years and may have influenced NT measurements to some extent. Third, we focused primarily on malformations and chromosomal abnormalities detected by karyotyping. Monogenic disorders and copy number variations were not systematically investigated, as comprehensive molecular genetic testing became widely accessible in Germany only in recent years, when it was included in statutory health insurance coverage. Therefore, undetected genetic contributions to adverse outcomes cannot be excluded. Fourth, this study is limited by the absence of multivariable regression analyses adjusting for potential confounders such as maternal age and gestational age. While NT measurement intrinsically accounts for gestational age, and analyses were performed with standardized protocols, residual confounding cannot be completely excluded. Finally, missing data for certain outcome variables, including malformations and pregnancy outcomes, may have limited the statistical power of some subgroup analyses.

## Conclusions

Our study demonstrates that NT measurements in fetuses with CRL measurements below 45 mm have predictive value for future outcomes. The presence of elevated NT in this population strongly indicates chromosomal abnormalities, structural malformations, and adverse pregnancy outcomes. The study results show that NT measurement delivers critical early diagnostic information despite current risk modeling restrictions for this specific CRL range. The use of genomic screening without anatomical imaging would result in a significant clinical risk because it would miss nearly one-third of all abnormalities. The detection of an increased NT at any gestational age before 11 weeks requires complete evaluation through expert ultrasound and genetic counseling and invasive diagnostic procedures when necessary. The management of NT measurements equal to or greater than 3.5 mm in fetuses with CRL less than 45 mm should follow the same diagnostic approach as standard CRL range measurements until new prospective data establishes alternative thresholds. The optimal prenatal care for this population requires early fetal medicine unit referrals together with first-trimester anatomical scans and genetic counselling.
